# Prevalence trends of hypertension and influence factors among children and adolescents aged 7–17 years in China, 2011–2015: A serial cross-sectional study

**DOI:** 10.3389/fpubh.2022.887285

**Published:** 2022-10-13

**Authors:** Yunjuan Yang, Jing Dai, Jieqing Min, Huamei Wu, Songquan Huang, Qingsheng Li, Jiajia Chai

**Affiliations:** ^1^Department of School Health, Yunnan Provincial Center for Disease Control and Prevention, Kunming, China; ^2^Public Health School, Kunming Medical University, Kunming, China; ^3^Public Health School, Dali University, Dali, China; ^4^Management and Economy School, Kunming University of Science and Technology, Kunming, China; ^5^Kunming Children's Hospital, Kunming, China; ^6^The Second Affiliated Hospital of Kunming Medical University, Kunming, China

**Keywords:** hypertension, children, adolescent, epidemic trend, prevalence, influence factors, cross-sectional survey

## Abstract

Hypertension has rapidly increased in the last decades throughout the world. It is an emerging disease. However, limited information is available on secular trends and factors of childhood and adolescents' hypertension in China. In this study, 5-year successive data were derived from a cross-sectional study of the China Health and Nutrition Survey (CHNS) in 2011 and 2015. We used systolic blood pressure (SBP) and/or diastolic blood pressure (DBP) at least 95th percentile on the basis of age, sex, and height percentiles to define hypertension (HBP). A total of 2,827 children and adolescents aged 7–17 years were included. The age-standardized prevalence of hypertension was increased significantly across 5 years: the standardized prevalence of hypertension was increased from 8.08% (2011) to 11.46% (2015) in China (*P* < 0.01). The mean SBP of boys increased from 101.21 to 102.79 mmHg, while the mean SBP of girls increased from 98.96 to 100.04 mmHg. The mean DBP of boys increased from 61.20 to 67.40 mmHg, while the mean DBP of girls increased from 64.34 to 65.76 mmHg. The prevalence of hypertension grew continuously in both sexes, but the pace of change for boys was more rapid than that for girls. This study confirmed that the association between rural (odds ratio [*OR*] = 1.394, 95%CI 1.032–1.883), overweight/obesity (*OR* = 2.621, 95%CI 1.506–4.562), and BP levels was highly correlated (*P* < 0.05). The possible protecting factors associated with hypertension were being a girl (*OR* = 0.788, 95%CI 0.595–1.043). There was no association between weekly physical activity, daily sleep duration, and hypertension (*P* > 0.05). Further in-depth analysis of influencing factors and comprehensive interventions should be urgently implemented to combat the hypertension epidemic among children and adolescents in China.

## Introduction

Hypertension is the most common chronic non-communicable disease. It is also a serious medical condition and a risk factor of atherosclerotic disease, stroke, diabetes, kidney failure, and blindness (1–3). It is a major cause of premature death worldwide, with upward of 1 in 4 men and 1 in 5 women over a billion people having the condition (4–7). According to the WHO report of the estimated 1.13 billion people who have hypertension, fewer than 1 in 5 have it under control (8).

With the development of social economy and the changing in people's lifestyles, once childhood and adolescents' hypertension were rare. At present, it has become a serious public health challenge (9, 10). The origin of hypertension in adulthood could be traced back to its presence in childhood (11–13). Children with elevated blood pressure are more likely to become hypertension adults (14, 15). Therefore, it is important to identify childhood hypertension timely.

This study aimed to estimate the prevalence trends of hypertension in children and adolescents over the recent 5 years in China, and explore the risk factors associated with it to inform effective strategies and measures in secondary prevention of hypertension in children and adolescents. This study used data from the China Health and Nutrition Surveys (CHNS) conducted during 2011–2015 for analysis.

## Methods

### Data

The data were derived from the CHNS, which were cross-sectional studies conducted in 2011 and 2015 (16). The general information, methods, and dataset information are available on the following website (http://www.cpc.unc.edu/projects/china). The sampling methods were a series of complex multistage stratified cluster sampling. In brief, the participants were sampled from 12 Chinese cities or provinces (i.e., Beijing, Liaoning, Heilongjiang, Shanghai, Jiangsu, Shandong, Henan, Hubei, Hunan, Guangxi, Guizhou, and Chongqing). The survey design and methods have been described in detail elsewhere (17, 18). The committees of the University of North Carolina and the Chinese Center for Disease Control and Prevention reviewed the ethical guidelines at Chapel Hill and approved the procedures for data collection, and all participants and/or their parents/guardians provided written informed consent.

This study was performed in line with the principles of the Declaration of Helsinki, and in accordance with all relevant guidelines and regulations for medical research. We collected data from 2,827 (1,461 boys and 1,366 girls) subjects aged 7–17 years in our study, namely, in 2011 (*n* = 1,468) and 2015 (*n* = 1,359).

### Measurements and definitions

All participants enrolled in the surveys underwent a physical examination to collect anthropometric data [weight, height, and blood pressure (BP)]. Weight was measured to the nearest 0.10 kg with a balance-beam scale, while the subjects were wearing lightweight clothing. Height was measured to the nearest 0.1 cm using a stadiometer according to a standardized protocol. Body mass index (BMI) was calculated as weight in kilograms divided by height in meters squared. BP was measured according to the recommendation of the National High Blood Pressure Education Program (NHBPEP) Working Group in Children and Adolescents (19), using an auscultation mercury sphygmomanometer by trained and qualified personnel who followed a standard protocol, as described elsewhere (20). BP was measured after resting for at least 5 min in the sitting position. An appropriately sized cutoff value was used to measure the BP of each child on their right arm. Systolic blood pressure (SBP) was determined by the onset of the first “tapping” Korotkoff sound (K1), and diastolic blood pressure (DBP) was determined by the fifth Korotkoff sound (K5). The mean of the three measurements was analyzed. Height is an identified important factor in the process of establishing pediatric hypertension (HBP) references worldwide (21, 22). Therefore, this study used the Chinese age-specific and height-specific BP references to classify the abnormal status of boys and girls separately (23).

Hypertension was defined as SBP and/or DBP at least 95th percentile on the basis of age, sex, and height percentiles (19, 23).

A questionnaire was used to collect demographic information, including physical activity, sleep time, cigarette smoking, and alcohol use. Data were collected by self-report in the CHNS, in 2011 and 2015. The sleep time was the students' average daily sleep time. Then, their sleep time status was categorized as “up to standard” and “not up to standard,” according to the standard sleeping time for primary and secondary school students developed by the Ministry of Education (at least 9 h/day). In addition, their physical activity was categorized by whether the students have moderate-intensity activity every week or not. Smoking status was categorized by whether they have smoked cigarettes in the past or not. Alcohol/beer use was categorized by whether they have drunk alcohol/beer in the past or not.

### Statistical analysis

Differences in the SBP and DBP of different survey years by age, gender, region, and area were compared using the ANOVA test. The prevalence of hypertension was calculated in different survey years (2011 and 2015) and further stratified by age, gender, region, and area (urban and rural). The division of urban and rural areas was based on the Chinese Administrative Division. Univariate methods were not adopted to estimate *P*-value for differences by sex, age, region, and area group, because high statistical power was achieved from the large sample sizes.

For comparability, the age-standardized prevalence was calculated by using the population of China Census 2010 as a standard population. The age groups were manually set into 3 groups, namely, 7–10 years, 11–14 years, and 15–17 years. Chi-square analyses were conducted to assess differences in the prevalence of hypertension for categorical variables.

Then, chi-square analyses had statistical different indicators as logistic analysis factors. Univariate and multivariate unconditional logistic regression analysis was used to analyze the risk factors of hypertension.

All data were analyzed using the IBM SPSS version 24.0 software. All analyses included sample weights and percentages of the unequal probabilities of selection, oversampling, and non-response. Statistical significance was defined as *P* < 0.05.

### Data availability statement

The datasets of this study are available from the corresponding author upon reasonable request.

## Results

### Characteristics of participants

A total of 2,827 school-aged children and adolescents were included in the surveys in 2011 and 2015. Among them, 364 were excluded for missing data or having extreme abnormal values of height, weight, and BP, and 2,463 were effective, with an effective rate of 87.12%. As shown in Table 1, there were 1,261 boys and 1,202 girls. No significant difference was found in the distribution of gender groups in 2011 and 2015. These data were comparable. The average age was 11.09 ± 2.94 years old. There were 868 urban and 1,595 rural participants. In addition, there were 829 from Eastern regions, 645 from Central regions, and 989 from Western regions. Significant differences were observed in the distribution of participants' age, area, and region groups in 2011 and 2015.

**Table 1:** The demographics of surveyed children, 2011–2015.

**Index**	**2011**	**2015**	***χ^2^***	***P***
**All**	1,405	1,058		
**Gender**			0.188	0.664
Boys	714	547		
Girls	691	511		
**Age**			31.236	0.000
7–10y	606	544		
11–14y	531	393		
15–17y	268	121		
**Region**			11.134	0.004
Eastern	505	324		
Central	337	308		
Western	563	426		
**Area**			9.334	0.002
Urban	531	337		
Rural	874	721		
**Usually does physical exercises**			0.632	0.729
No	852	642		
Yes	538	401		
No answer	15	15		
**Ever smoke cigarettes**			0.140	0.933
No	644	355		
Yes	8	8		
No answer	753	695		
**Drinking alcohol/beer last year**				
No	605	113	0.197	0.906
Yes	47	14		
No answer	753	931		
**BMI group**			2.420	0.298
Thin	169	121		
Normal	956	699		
Overweight and obesity	280	238		

Furthermore, 38.12% of participants had regular exercises, and 21.03% of them had overweight and obesity in the surveys. No significant difference was found in the distribution of the number by doing exercises and BMI group in 2011 and 2015. These data were comparable.

### Secular changes in BP

**Figure 1:**
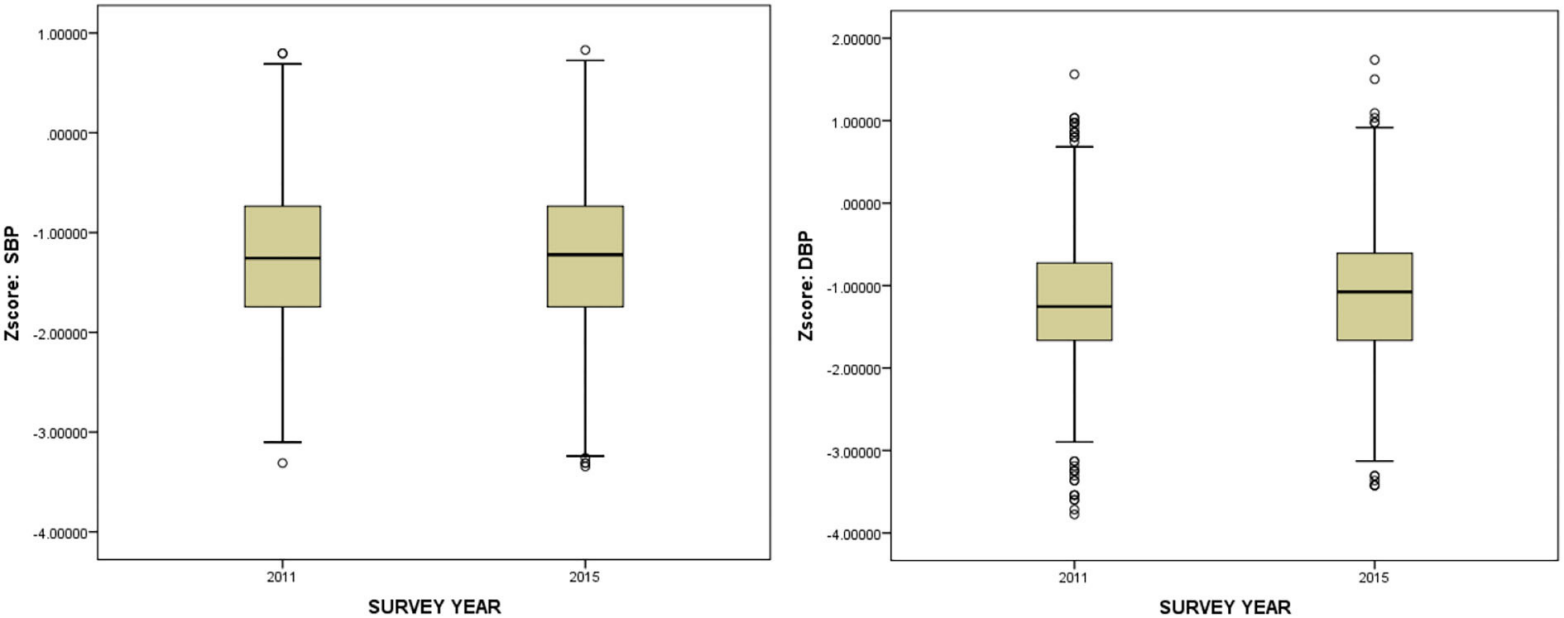
Overall trend of standardized blood pressure (SBP and DBP) in subjects aged 7–17 years in China, 2011–2015.

As shown in Figure 1, the increase in the overall standardized DBP trend was more significant than that in standardized SBP from 2011 to 2015.

**Figure 2:**
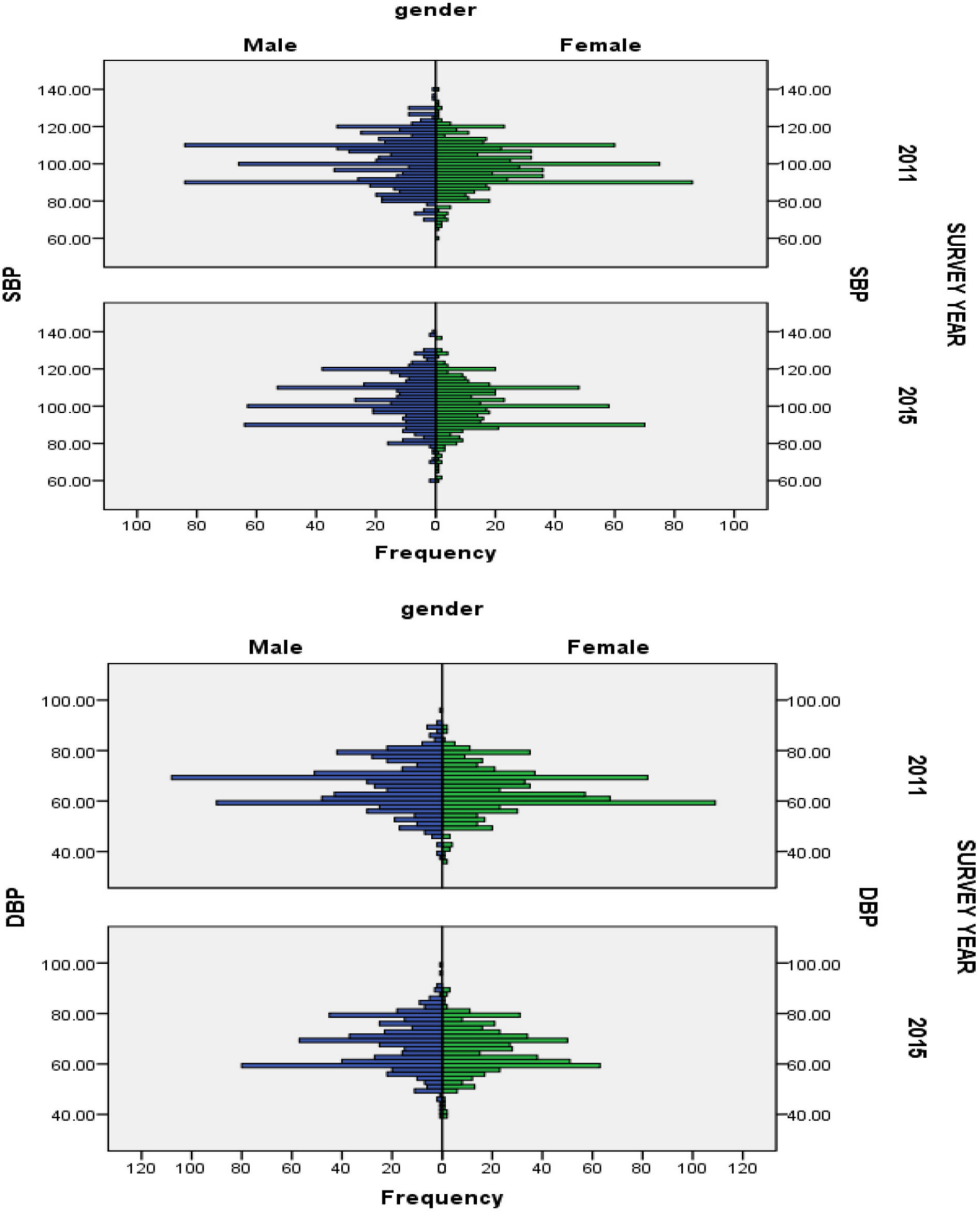
Secular trend of blood pressure distribution (SBP and DBP) in different gender aged 7–17 years in China, 2011–2015.

As shown in Figure 2 in both boys and girls, the distribution of blood pressure (SBP and DBP) of the high value increased from 2011 to 2015, and that of the middle value decreased obviously.

In 2011 and 2015, the mean SBP of boys increased from 101.21 to 102.79 mmHg, while it increased from 98.96 to 100.04 mmHg for girls; the mean DBP of boys increased from 61.20 to 67.40 mmHg, while it increased from 64.34 to 65.76 mmHg for girls (Table 2). Compared with girls, the mean BP for boys increased slightly faster in each age group. Similar increasing trends were observed across all age groups in both genders (*P* < 0.05).

**Table 2:** The characteristics of blood pressure among different age and gender, 2011–2015.

**Subgroup**	**SBP**		***F***	**DBP**		***F***	**Zscore: SBP**		***F***	**Zscore: DBP**		***F***
	**2011**	**2015**		**2011**	**2015**		**2011**	**2015**		**2011**	**2015**	
**Boys**			4.600*			3.891**			4.600*			3.891**
7–10y	95.31 ± 11.62	99.39 ± 12.49		62.11 ± 8.73	66.01 ± 9.44		−0.43 ± 0.93	−0.10 ± 0.99		−0.41 ± 0.96	0.01 ± 1.03	
11–14y	102.89 ± 11.88	103.86 ± 12.41		61.95 ± 8.72	67.40 ± 9.73		0.18 ± 0.95	0.25 ± 0.99		0.22 ± 0.95	0.17 ± 1.06	
15–17y	110.94 ± 10.95	113.97 ± 9.99		72.50 ± 7.96	73.39 ± 7.18		0.82 ± 0.87	1.06 ± 0.80		0.72 ± 0.87	0.82 ± 0.79	
Total	102.21 ± 12.95	102.79 ± 13.01		61.20 ± 8.79	67.40 ± 9.58		0.04 ± 1.03	0.17 ± 1.04		0.05 ± 1.03	0.17 ± 1.05	
**Girls**			2.404			8.031*			2.404			8.031*
7–10y	93.72 ± 11.30	96.95 ± 12.23		61.20 ± 8.79	64.33 ± 8.54		0.56 ± 0.90	−0.30 ± 0.97		−0.51 ± 0.96	−0.17 ± 0.93	
11–14y	101.90 ± 10.37	102.15 ± 11.47		66.06 ± 7.75	66.64 ± 8.41		0.10 ± 0.83	0.12 ± 0.91		0.02 ± 0.85	0.08 ± 0.92	
15–17y	105.39 ± 9.89	107.60 ± 9.76		68.22 ± 7.08	69.58 ± 8.39		0.37 ± 0.79	0.55 ± 0.78		0.25 ± 0.77	0.40 ± 0.92	
Total	98.96 ± 11.73	100.04 ± 12.22		64.34 ± 8.60	65.76 ± 8.63		−0.14 ± 0.93	−0.05 ± 0.97		−0.17 ± 0.94	−0.01 ± 0.94	

*P < 0.05; ** *P* < 0.01.

### The prevalence of hypertension

The crude prevalence of hypertension was 9.74% (240/2,463) in children and adolescents aged 7–17 years in China. The standardized prevalence was 9.54%.

In 2011 and 2015, the crude prevalence of hypertension among children and adolescents aged 7–17 years was 8.04% (113/1,405) and 12.00% (127/1,058), respectively, in China (χ^2^ = 10.767, *P* < 0.01). After standardization, the prevalence increased from 8.08% in 2011 to 11.46% in 2015 in China. The average annual increase (AAI) rate was 0.68%.

**Figure 3:**
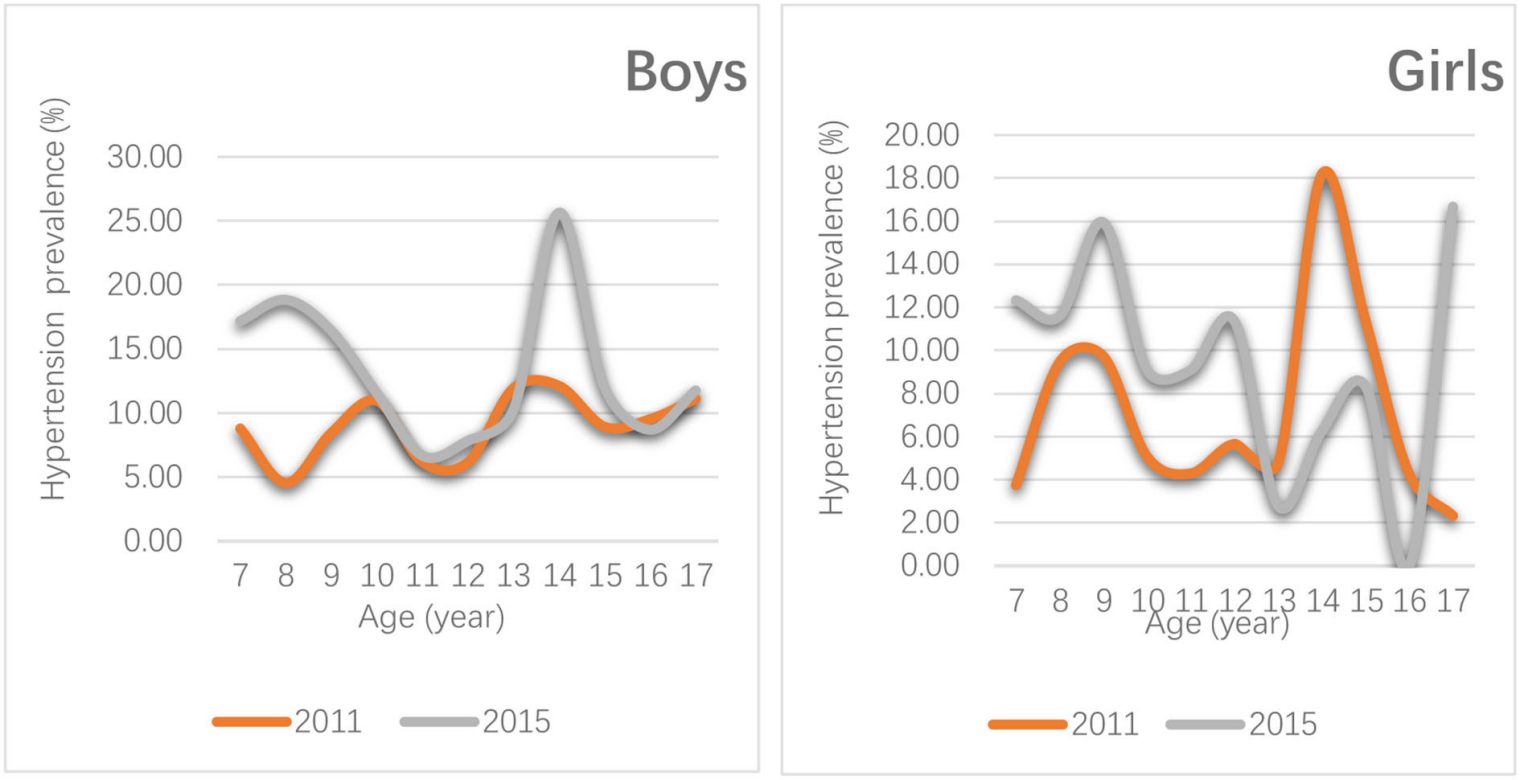
Distribution change of the prevalence of hypertension in boys and girls aged 7–17 years in China, 2011–2015.

The crude prevalence of hypertension for boys was 8.82% (63/714) and 13.53% (74/547), respectively, in China (χ^2^ = 7.079, *P* < 0.01). The prevalence of hypertension for girls was 7.24% (50/691) and 10.37% (53/511), respectively, in China (χ^2^ = 3.687, *P*>0.05). The standardized prevalence of hypertension for boys and girls was 9.33% (2011) and 13.18% (2015) and 7.09% (2011) and 9.12% (2015), respectively. As shown in Figure 3, the prevalence of hypertension for boys increases faster. The AAI rate was 0.81%. A significant difference was observed in the prevalence of different gender groups in 2011 and 2015 (χ^2^ = 3.700, *P* = 0.05).

In different regions, the prevalence of hypertension was 8.12% (41/505) and 15.43% (50/324) among children and adolescents, respectively, in Eastern China (χ^2^ = 10.802, *P* < 0.01). A significant difference was observed in the prevalence in different years in Eastern population. The prevalence of hypertension in Eastern region increased faster, with the AAI rate of 1.46%. In the Central China, the prevalence was 9.50% (32/337) and 12.01% (37/308), respectively, in 2011 and 2015 (χ ^2^ =1.068, *P*>0.05) and that in the Western China was 7.10% (40/563) and 9.39% (40/426), respectively, in 2011 and 2015 (χ^2^ = 1.703, *P* > 0.05). A significant difference was observed in the prevalence of different region groups in 2015 (χ^2^ = 6.360, *P* < 0.05).

As shown in Table 3, with the increase in BMI, the prevalence of hypertension in children and adolescents aged 7–17 years in China was also increased. In 2011 and 2015, the prevalence changes of hypertension with different BMI types were particularly significant in emaciation, overweight, and obesity groups. Over 5 years, the AAI rate was 1.11% in emaciated children and adolescents. The AAI rate was 1.50% in overweighed and obese children and adolescents. In addition, the prevalence level of hypertension was also statistically significant with self-perceived body weight. With the increase in self-perceived body weight, the prevalence of hypertension was also increased. Over 5 years, the AAI rate was 0.89% in self-perceived weighed normal children and adolescents.

**Table 3:** Trends in the prevalence of hypertension among children and adolescents aged 7–17 years in China, 2011–2015.

**Index**	**2011**			**2015**			**AAI(%)**	***χ^2^***	***P***
	**N**	**hypertension**	**%**	**N**	**hypertension**	**%**			
**All**	1,405	113	8.04	1,058	127	12.00	0.79		
**Gender**								3.701	0.054
Boys	714	63	8.82	547	74	13.53	0.94		
Girls	691	50	7.24	511	53	10.37	0.63		
**Age**								2.335	0.311
7–10y	606	46	7.59	544	77	14.15	1.31		
11–14y	531	46	8.66	393	38	9.67	0.20		
15–17y	268	21	7.84	121	12	9.92	0.42		
**Region**								5.180	0.075
Eastern	505	41	8.12	324	50	15.43	1.46		
Central	337	32	9.50	308	37	12.01	0.50		
Western	563	40	7.10	426	40	9.39	0.46		
**Area**								3.201	0.074
Urban	531	35	6.59	337	37	10.98	0.88		
Rural	874	78	8.92	721	90	12.48	0.71		
**Usually does physical exercises**								0.057	0.811
No	852	74	8.69	642	72	11.21	0.50		
Yes	538	38	7.06	401	51	12.72	1.13		
No answer	15	1	6.67	15	4	26.67	4.00		
**Ever smoke cigarettes**								10.767	0.001
No	644	59	9.16	355	38	3.52	−1.13		
Yes	8	1	12.50	8	1	12.50	0.00		
No answer	753	53	7.04	695	88	12.66	1.12		
**Drinking alcohol/beer last year**								10.767	0.001
No	605	55	9.09	113	12	10.62	0.31		
Yes	47	5	10.64	14	1	7.14	−0.70		
No answer	753	53	7.04	931	114	12.24	1.04		
**BMI group**								22.015	0.000
Emaciation	169	6	3.55	121	11	9.09	1.11		
Normal	956	75	7.85	699	71	10.16	0.46		
Overweight and obesity	280	32	11.43	238	45	18.91	1.50		
**Self-feeling weight**								7.059	0.029
Low weight	275	18	6.55	163	17	10.43	0.78		
Normal	851	62	7.29	697	82	11.76	0.89		
Overweight	216	27	12.50	128	19	14.84	0.47		
Unknown	63	6	9.52	70	9	12.86	0.67		
**Sleep time**								0.121	0.728
Up to standard	825	63	7.64	576	64	12.57	0.99		
Not up to standard	509	50	8.68	541	61	11.28	0.52		

As shown in Table 3, there was no statistical significance in weekly physical activity and daily sleep duration with the prevalence of hypertension.

As shown in Figure 3, the age with the fastest rise in the prevalence of hypertension was 13–15 years (boys), 7–10 years (boys), and 7–12 years (girls) in 2011 and 2015.

### Logistic regression analyses

The independent factors associated with hypertension were urban or rural (*P* < 0.05), and overweight and obesity (*P* < 0.01). The possible protecting factors associated with hypertension were being a girl (*P*>0.05) (Table 4).

**Table 4:** Multivariate analysis with logistic regression.

**Variables**	**β**	**S.E**.	**Odds ratio**	**95%CI**	***P*-value**
Gender	−0.239	0.312	0.788	0.595–1.043	0.096
Urban or rural	0.332	0.153	1.394	1.032–1.883	0.031
Emaciation	0.413	0.267	1.511	0.896–2.550	0.122
Overweight and obesity	0.963	0.283	2.621	1.506–4.562	0.001
Constant	−2.858	0.286	0.057		

## Discussion

In this study, our results indicated that the standardized prevalence of hypertension was increased significantly from 8.08 to 11.46% from 2011 to 2015, especially in boys in China (9.33% in 2011 to 13.18% in 2015). This increase was more rapid during 2011 and 2015. This study also confirmed that being a girl was the possible protecting factors associated with hypertension (*OR* = 0.788, 95%CI 0.595–1.043). This study indicated that the AAI of hypertension was 0.79%. Compared with previous reports that analyzed the same national survey data, this study reported a similar increasing trend (20, 24) but at a faster-increasing speed, as shown by the AAIs of the hypertension [0.33% (25); 0.19% (26)]. This difference can be partly explained by the differences in methodology as this study excludes extreme abnormal values of height and BP and uses age-specific and height-specific BP references to classify hypertension, whereas the previous study has only used national age-specific BP cutoff values (25) or has not excluded abnormal values (26).

The standardized prevalence of hypertension among children and adolescents aged 7–17 years in China (11.46% in 2015) was higher than that in Korean (9.0%), US (1.6%), Brazil (4.5%), and Cameroon (1.6%) levels (27–30). This study finds a warning that the hypertension epidemic is accelerating among children and adolescents aged 7–17 years in China. The problem of children and adolescents' hypertension should be paid strong attention. Previous studies have confirmed that childhood hypertension not only affected children's health but also had a “trajectory” of BP. That is, childhood hypertension can gradually develop into adult hypertension and can increase the risk of cardiovascular disease in adulthood (3, 10, 11, 15, 23, 31). Therefore, we should pay more attention focusing on children and adolescents' hypertension prevention and control, especially in boys.

In addition, logistic regression analysis results indicated that the prevalence of hypertension of rural children and adolescents was higher than that of the urban population (*OR* = 1.394, 95%CI 1.032–1.883). Rural children and adolescents were 1.394 times more likely to have hypertension than urban children and adolescents. This finding is consistent with the previous research (26). It suggests that health education should carry out interventions to target children's hypertension in rural areas.

Furthermore, this study confirmed that the association between overweight/obesity and BP levels was strong (*OR* = 2.621, 95%CI 1.506–4.562). Overweight/obesity was the independent risk factor of children and adolescents' hypertension (*P* < 0.01). This result has been reported in previous studies (32, 33). In addition, the consistent increasing trends in the overweight/obesity and the prevalence of high hypertension in rural boys compared with boys in urban strongly support that the obesity epidemic remains an important contributor to the increasing trend in hypertension among children and adolescents aged 7–17 years in China. BMI can be used as an independent predictor of hypertension in children and adolescents. Some studies had also confirmed that controlling BMI is more effective in reducing the risk of hypertension (34–36). Therefore, it is suggested that publicity and education on the prevention and control of overweight and obesity should be widely carried out among children and adolescents to advocate a healthy lifestyle, especially to promote physical exercise and a reasonable diet. We shall strengthen the screening and intervention of early overweight and obesity in early childhood and regularly monitor weight and other indicators on obesity in children and adolescents, so as to achieve early prevention, early detection, and early control of overweight and hypertension in children and adolescents. At the same time, we should monitor what kind of role BMI changes can have in determining the onset of hypertension over time.

This study results indicated that there was no association between weekly physical activity, daily sleep duration, and hypertension (37–40). This can be partly explained when there was no significant difference between 2011 survey children and 2015. For example, there were 41.00% (576/1,405) of children in 2011 and 42.72% (452/1,058) of children in 2015, respectively, participating in daily activities. The prevalence of hypertension was 9.52 and 9.94% in adequate sleep children and insufficient sleep children, respectively. These findings should be confirmed by further research.

This study results may underestimate the prevalence of hypertension among school children and adolescents aged 7–17 years as the hypertension was defined as SBP and/or DBP at least 95th percentile based on age, sex, and height percentiles. Although weight-for-height is not a perfect measure of body fat and BP, it is highly correlated with body fat at the higher values (19). The study explored relative changes in the prevalence of hypertension of school-aged children and adolescents. Regardless of what measures were used, the trend was apparent. Then, this study used two cross-sectional surveys' data, as each survey was conducted on different subjects every 5 years. It was possible that unintentional errors occurred when estimating the prevalence of hypertension. Finally, this study can roughly find the impact of smoking and alcohol drinking on children and adolescents' hypertension. These indicators cannot be ignored. However, due to the sensitivity and privacy of smoking and alcohol use, the rate of no-response was too high, resulting in excessive bias of data. It was suggested that the effect of these indicators on children and adolescents' blood pressure needs to be paid close attention in future studies.

To have comparability between 2011 and 2015, the crude rates of the prevalence of hypertension were standardized according to the age distribution of the National Population Data of China Census 2010. This was critical to the reliability of the conclusion. Despite these limitations, our findings have a significant contribution to understand the epidemic level and secular trends of school children and adolescents aged 7–17 years in China.

In conclusion, this serial cross-sectional study indicated significant increasing trends in the prevalence of hypertension among children and adolescents aged 7–17 years in China from 2011 to 2015, especially in rural boys. Considering that childhood and adolescents' hypertension can develop into adult hypertension and increase the risk of cardiovascular disease, childhood and adolescents' hypertension should be a major public health threat. We should make further study to explore related risk factors to make a comprehensive intervention for preventing and controlling hypertension among children and adolescents in China.

## Data availability statement

The datasets presented in this study can be found in online repositories. The names of the repository/repositories and accession number(s) can be found in the article/supplementary material.

## Ethics statement

The studies involving human participants were reviewed and approved by the Committees of the University of North Carolina and the Chinese Center for Disease Control and Prevention reviewed the ethical at Chapel Hill and approved the procedures for data collection. Written informed consent to participate in this study was provided by the participants' legal guardian/next of kin. Written informed consent was obtained from the individual(s), and minor(s)' legal guardian/next of kin, for the publication of any potentially identifiable images or data included in this article.

## Author contributions

YJY, JD, and JQM conducted data collection, data check, statistical analysis, and interpretation. YJY conducted manuscript design and writing. HMW, SQH, and QSL advised on statistical analysis and some suggestions. JJC provided some suggestion, assisted in translation, and edited the final version of the manuscript. The corresponding author had full access to all the data in this study and took the primary responsibility for the final content. All authors read and approved the final version of the manuscript.

## Funding

This study was supported by the Natural Science Foundation of China (Grant No. 71764014), the Yunnan Provincial Grant for the Academic Leadership in Medical Science (Grant No. D-2018007), the 16th Batch of Kunming Grant for the Young Academic and Technical Leadership (Grant No. KMRCD-2018011), and a grant by the Xishan District Bureau of Science and Technology (Grant No. 34 Xikezi).

## Conflict of interest

The authors declare that the research was conducted in the absence of any commercial or financial relationships that could be construed as a potential conflict of interest.

## Publisher's note

All claims expressed in this article are solely those of the authors and do not necessarily represent those of their affiliated organizations, or those of the publisher, the editors and the reviewers. Any product that may be evaluated in this article, or claim that may be made by its manufacturer, is not guaranteed or endorsed by the publisher.
